# Red cell adenylate kinase deficiency in China: molecular study of 2 new mutations (413G > A, 223dupA)

**DOI:** 10.1186/s12920-022-01248-2

**Published:** 2022-05-04

**Authors:** Sijia He, Hongbo Chen, Xia Guo, Ju Gao

**Affiliations:** 1 Department of Peadiatrics, West China Second University Hospital, Sichuan University, Chengdu, 610041 China; 2 Key Laboratory of Birth Defects and Related Diseases of Women and Children, Sichuan University, Ministry of Education, Chengdu, 610041 China

**Keywords:** Adenylate kinase, AK deficiency, Haemolytic anaemia, Next-generation sequencing

## Abstract

**Background:**

Adenylate kinase (AK) is a monomolecular enzyme widely found in a variety of organisms. It mainly catalyses the reversible transfer of adenosine nucleotide phosphate groups and plays an important role in maintaining energy metabolism. AK deficiency is a rare genetic disorder that is related to haemolytic anaemia. Chronic haemolytic anaemia associated with AK deficiency is a rare condition, and only 14 unrelated families have been reported thus far. Moreover, only 11 mutations have been identified in the *AK1* gene*,* with only 3 cases of psychomotor impairment.

**Case presentation:**

The patient was a 3-year-old boy with severe haemolytic anaemia and psychomotor retardation. A molecular study of the patient’s *AK* gene revealed 2 different mutations: a heterozygous missense mutation in exon 6 (c.413G > A) and a heterozygous frameshift mutation in exon 5 (c.223dupA). Molecular modelling analyses indicated that AK gene inactivation resulted in a lack of AK activity. The patient recovered after regular blood transfusion therapy.

**Conclusions:**

AK1 deficiency was diagnosed on the basis of low enzymatic activity and the identification of a mutation in the *AK1* gene located on chromosome 9q. Here, we report the first case of moderate red cell AK1 deficiency associated with chronic nonspherocytic haemolytic anaemia (CNSHA) in China. The genetic mutations were confirmed by Sanger sequencing. The variants were classified as pathogenic by bioinformatics tools, such as ACMG/AMP guidelines, Mutation Taster, SIFT, MACP, REVEL and PolyPhen2.2. Based on our evidence and previous literature reports, we speculate that the site of the *AK1* gene c.413G > A (p.Arg138His) mutation may be a high-frequency mutation site and the other mutation (c.223dupA) might be related to the neuropathogenicity caused by AK1 deficiency. NGS should be a part of newborn to early childhood screening to diagnose rare and poorly diagnosed genetic diseases as early as possible.

**Supplementary Information:**

The online version contains supplementary material available at 10.1186/s12920-022-01248-2.

## Background

Adenylate kinase (AK) is a monomolecular enzyme that is widely found in a variety of organisms. Its main role is to catalyse the reversible transfer of adenosine nucleotide phosphate groups, with an important role in maintaining the energy metabolism of cells [1, 2]. The molecular formula is ATP∙Mg^2+^  + AMP ⇋ ADP· Mg^2+^  + ADP, and Mg^2+^ plays an electrostatic barrier role to promote the production of catalytic substrates [3, 4]. The AK family contains proteins with one or two catalytic domains. This family of proteins is present in red blood cells and muscle and exists as 9 different isozymes designated AK1 to AK9 [5, 6]. The human erythrocytic isoenzyme AK1 represents the major cytosolic isoform and is encoded by a 12 kb long gene (*AK-1*) [7]. AK1 is a small cytosolic enzyme, and the highest expression levels occur in the skeletal muscle, the brain and erythrocytes [8]. The gene encoding *AK1* is located at 9q34 and contains seven exons that produce a 194 amino acid polypeptide. AK1 deficiency is a rare genetic disorder that has been related to haemolytic anaemia. To date, only 14 cases of AK1 deficiency have been reported in unrelated families distributed worldwide [9].

In this paper, we report the first case of moderate red cell AK1 deficiency associated with chronic nonspherocytic haemolytic anaemia (CNSHA) in China. The patient was a 3-year-old boy born of a nonconsanguineous marriage. The patient carried a compound heterozygous mutation in the *AK1* gene and presented with intellectual disability and severe haemolytic anaemia. A molecular study of the patient’s *AK1* gene revealed 2 different mutations. Since AK1 deficiency is inherited in an autosomal recessive pattern, the patient’s family was also screened for *AK1* gene mutations.

## Case presentation

### Clinical reports

The boy, aged 3 years and 10 months, was a full-term child with no history of asphyxia at birth. He was hospitalized in another hospital due to "severe neonatal hyperbilirubinemia" as a newborn. Routine blood examination during this period showed Hb 129 g/L, HCT 39.2%, MCV 118.8 fl, MCH 39.1 pg, MCHC 329 g/L, and RET % 2.48%. The biochemistry revealed TB 516 µmol/L and IDIL 484 µmol/L. Finally, he was improved after blood exchange therapy. When he was 3 years old, he was found to have haemolytic anaemia (Hb 59 g/L, Ret% 11.3%, Ret 0.83*10^12^/L, TB 77.2 µmol/L, and LDH 3392 U/L). Bone marrow examination revealed hyperplastic anaemic bone marrow, and the ratio of granulocytes to red blood cells was 0.12:1. According to the patient's early age of onset and related clinical manifestations, the most common enzymes that cause haemolytic anaemia, such as G6PD, PK and P5′N, were routinely measured, However, they were found to be in the normal range (Table 1). After regular blood transfusion treatment in the Outpatient Department of our hospital, his Hb gradually increasead to approximately 100 g/L. Intellectual disability was later reported after the patient had completed EEG and MRI, which showed normal results. The patient was then tested by the Chinese Wechsler Young Children scale of intelligence (C-WYCSI), which revealed a low value (Table 1). The C-WYCSI is an internationally recognized and universal intelligence test for children aged 6–16. When the age is less than 18 years old and the IQ is less than 70, the retardation of intellectual development is considered. A value of 50–69 is mild, 35–49 is moderate, 20–34 is severe, and 0–19 is very severe. The C-WYCSI test suggested mild intellectual disability.

**Table 1:** Haematological, biochemical, molecular data of the patient and his parents

Haematological	Neonatal period	Preschool period	Father	Mother	Reference ranges
Age/sex	4D	3/M	31Y	31Y	
WBC	6.1	7	6.61	6.14	4–10 * 10^9^/L
RBC	2.12	2.46	4.99	4.49	3–5 * 10^9^/L
NEUT%	55.2	45.3	44	45	5–50%
LYMPH%	45.8	47.7	66	65	30–80%
HGB	129	64	146	128	100–145 * 10^9^/L
HCT	39.2	20.1	44.5	40.4	37–50%
MCV	118.8	81.7	89	89.9	80-90 fl
MCH	39.1	26	29.2	28.5	23-30 pg
MCHC	329	318	328	317	304–360 g/L
IRF	55.7	54.5	56	55	
RET%	2.48	7.48	0.8	1.3	0.5–1.5%
RET#	0.191	0.184	0.08	0.09	0.024–0.084 * 10^12^/L
PLT	306	184	321	341	150–540 * 10^9^/L
*Biochemical*					
ALT	35	31			0–49 U/L
AST	29	44			0–40 U/L
TB	516	31.7			5–20 umol/L
DBIL	32	12.6			0–6.8 umol/L
IDIL	484	19.1			0–17 umol/L
LDH	461	477			120–250 U/L
ALP	276	282			125–250 U/L
G6PD		2745u/gHG			
SI		22.47			11–30 umol/L
Tf		2.66			2.1–3.6 g/L
TIBC		57.9			44–69 umol/L
TS		39			20–55%
VitB12		176			18–670 pmol/L
FOL		> 54			> 12.2 nmol/L
SF		497.9			22–322 ng/mL
AK (200–250 IU/gHb)		184	202	209	200–250 IU/gHb
PK (5–15 IU/gHb)		6	9	11	5–15 IU/gHb
P5′N ratio(2.5–4)		3.1	3.5	2.8	2.5–4
*Other*					
Osmotic fragility of RBC		Neg			
Coombs test		Neg			
Thalassaemia test		Neg			
*Chinese Wechsler Young Children scale of Intelligence (C-WYCSI)*					
Language test		60			
Operation test		52			
Full scale intelligence quotient		52			
Intelligence quotient		52			

### Molecular genetic analysis

**Fig. 1:**
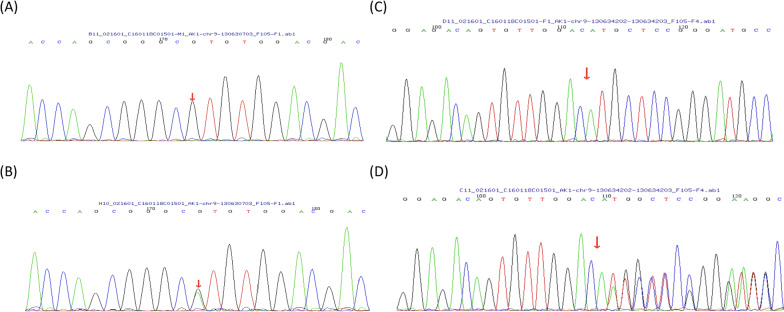
Sequence electropherograms of the *AK1* gene of exon 6 wild- and mutant-type c.413G > A changing codon p.Arg138His (**A**, **B**) and shows exon 5 wild- and mutant-type c.223dupA changing p.M75fs (**C**, **D**)

**Fig. 2:**
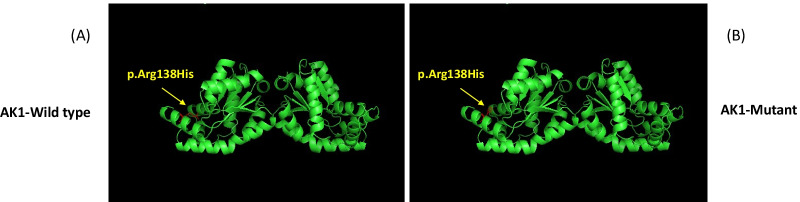
SWISS-MODEL-predicted structures of human AK1WT and AK1R138H mutants. The structures of the proteins are shown as cartoons. The important residues for the changed amino acids are shown as sticks. **A** Wild-type AK1 (yellow arrow); **B** Mutant-type p.Arg138His shows diminished hydrogen bonding (yellow arrow) due to glutamate

We further used a custom gene panel to perform next-generation sequencing. Genomic DNA of the proband was extracted from whole blood using a DNA Extraction kit (TIANGEN, Beijing, China) according to the manufacturer’s instructions. The DNA was quantified using a Nanodrop 2000 (Thermo Fisher Scientific, Waltham, MA, USA). Genomic DNA (3 μg) was fragmented by nebulization. The fragmented DNA was end-repaired and A-tailed using standard protocols. The size-selected product was PCR amplified, and the final product was validated using an Agilent Bioanalyzer (Agilent Technologies, Santa Clara, CA, USA). A total of 283 genes were captured using a GenCap custom enrichment kit (MyGenostics, Beijing, China) (Additional file 1: Table S1). The gene panel that codes for RBC enzymes and RBC membrane proteins and congenital dyserythropoietic anaemia was used [10, 11]. The genome sequence reference version was derived from the human reference genome (GRCh37/hg19). The custom panels for HAs that we used can provide diagnostic yields of 26.63%. The enriched libraries were sequenced using an Illumina HiSeq 2000 sequencer (Illumina, San Diego, CA, USA) using 100-base pair paired-end reads. All the suspected AK-causing mutations found by NGS were confirmed by Sanger sequencing. DNA sequences were obtained from the University of California Santa Cruz (UCSC) Genome Browser. The American College of Medical Genetics and Genomics (ACMG)/Association for Molecular Pathology (AMP) clinical variant interpretation guidelines established the criteria for the different types of evidence [12]. According to the ACMG/AMP Guidelines, this missense mutation was classified as a pathogenic variation (PVS1 + PM1_moderate + PM2_moderate + PP3). A null variant (frameshift) was confirmed in the gene for which loss of function is a known mechanism of disease (PVS1). This variant was located in a mutational hot spot and/or critical and well-established functional domain without benign variation (PM1). This variant was absent from controls (or at extremely low frequency if recessive) in the Exome Sequencing Project, 1000 Genomes Project, and Exome Aggregation Consortium (PM2) (Table 2). Both the missense mutation and frameshift mutation were classified as a disease causing by MutationTaster (http://www.mutationtaster.org/). The effect of the variant was evaluated by using PolyPhen2.2 (http://genetics.bwh.harvard.edu/pph2/), SIFT (http://sift.jcvi.org/), MACP and REVEL (https://sites.goolgle.com/site/revelgenomics/) to predict the pathogenic potential of the variants. (Table 2). To better understand the structural implications of the missense mutation identified in the human AK-1 protein, we have used UniProt (http://www.UniProt.org/) and SWISSMODEL (https://www.swissmodel.expasy.org) to build a three-dimensional model of the mutant protein. We downloaded the FASTA sequence of AK1 (ID: P00568-1) from UniProt and obtained mutation sequences from Mutalyzer. We selected 1z83.2 as the AK1 protein structure template and used the computer program MODELLER for automated modelling. The protein structures were visualized with PyMOL 1.7.4. Table 1 summarizes all the details of the haematological and biochemical studies conducted at West China Second University Hospital. AK enzyme activity detection showed a decreased level in red cells. This type of double heterozygous mutation has not yet been reported: a heterozygous missense variation in exon 6 (c.413G > A) that results in amino acid substitution of histidine for arginine at codon 138 (p.Arg138His) and a heterozygous frameshift mutation in exon 5 (c.223dupA) that results in an amino acid methionine position change at codon 75 (p.Met75Asnfs*19). Figure 1 shows the sequence electropherograms of the *AK1* exon 6 wild-type and mutant (A, B) and *AK1* exon 5 wild-type and mutant (C, D). To investigate the effect of heterozygous missense variation on the structure and function of the enzyme, molecular modelling analyses were conducted by using bioinformatics software, such as UniProt, SWISSMODEL and PyMOL 1.7.4. The structures of the human *AK1*^WT^ and *AK1*^R138H^ mutants are shown as cartoons and are labelled as wild-type p.Arg138His (A) and mutant-type p.Arg138His (B), respectively (Fig. 2). The patient exhibiting this compound heterozygous mutation was severely affected, whereas the patient’s parents, who were carriers for one of the mutations, were asymptomatic (Fig. 1).

**Table 2:** Exome sequence variants filtered by database and the prediction by ACMG/AMP guidelines

Chromosome location	Chr9-130630703	Chr9-130634202–130634203
c.variants	c.413G > A	c.223dupA
p.variants	p.R138H	p.M75fs
PolyPhen2 prediction (score)	Probably damaging (0.999)	NA
SIFT prediction (score)	Toleranted (0.06)	NA
MACP	P (0.353)	NA
REVEL	D (0.747)	NA
MutationTaster prediction	Disease causing	Disease causing
InterVar	Pathogenic	
PVS1	1	
PM1	1	
PM2	1	
PP3	1

## Discussion and conclusions

Chronic haemolytic anaemia associated with AK deficiency is a rare condition, and only 14 unrelated families have been reported thus far. To date, only 9 cases have been reported with 11 specific mutation types, including 2 heterozygous mutations [9, 13–19] (Table 3). We further confirmed this finding by identifying mutations via NGS, revealing a new type of double heterozygous mutation that has not yet been reported. *AK1* gene mutations can reduce the catalytic activity of type I adenosine kinase and destroy the stability of the protein, thus causing haemolytic anaemia [20]. AK deficiency is transmitted in an autosomal recessive mode of inheritance, and the heterozygous state may not be accompanied by disease [17]. To date, only 5 types of mutations have been identified in the *AK1* gene; these mutation types include missense, nonsense, frameshift, deletion and substitution mutations in 10 different families [21] (Table 3). Rashmi et al. [19] first used a disease-targeted NGS panel to detect mutations in the *AK1* gene to provide prenatal diagnosis. Only 3 cases of anaemia and psychomotor retardation have been reported, and AK activity could not be detected [13].

**Table 3:** List of all other human mutations cases already described in the literature and the mutation case detected in Chinese patient in *AK1* gene

No	Sex	Age (years)	Type of mutation	Nucleotide change	Amino acid change	Exons	Origin	References
1 (1989)	NA	NA	Missense	382C > T	Arg128Trp	Exon 6	Japan	Matsuura et al. [14]
2 (1999)	M	7	Nonsense	319C > T	Arg107Stop	Exon 5	Italy	Bianchi et al. [15]
3 (1997)	F	9	Missense	491A > G	Tyr164Cys	Exon 6	Italy	Qualtieri et al. [16]
4 (2003)	M	11 (m)	Missense	118G > A	Gly40Arg	Exon 4	Spain	Corrons et al. [17]
4 (2003)	M	11 (m)	Missense	190G > A	Gly64Arg	Exon 4	Spain	Corrons et al. [17]
5 (2003)	M	2	Deletion	418_420delGAC	DeletionD140	Exon 6	English	Corrons et al. [17]
6 (2004)	F	3	Frameshift	138delG	Glu46del	Exon 4	Italy	Fermo et al. [9]
7 (2017)	F	6	Missense	289C > T	Arg97Trp	Exon 5	Japan	Niizuma et al. [18]
8 (2019)	M	6	Missense	71A > G	Premature stop	Exon 4	India	Dongerdiye et al. [13]
8 (2019)	M	6	Missense	413G > A	Arg138His	Exon 6	India	Dongerdiye et al. [13]
9 (2021)	M	5	Substitution	301C > A	Gln101Lys	Exon 5	India	Dongerdiye et al. [19]
10 (2021)	M	3	Missense	413G > A	Arg138His	Exon 6	China	This paper
10 (2021)	M	3	Frameshift	223dupA	Met75Asnfs*19	Exon 5	China	This paper

The child's complete erythrocyte *AK-1* cDNA sequence was compound heterozygous for two novel mutations in the Chinese population, and cellular and molecular functional studied were carried out to confirm the pathophysiologic effect of the identified mutation. To further understand the cause of severe haemolytic anaemia, next-generation sequencing of 238 targeted genes associated with haemolytic anaemia was performed. One missense mutation (c.413G > A) and one frameshift mutation (c.223dupA) in the *AK1* gene were detected. To investigate how these mutations could lead to dysfunction of the enzyme molecule, we examined the phenotype correlations with three-dimensional structure analysis by UniProt, SWISSMODEL and PyMOL 1.7.4 [22, 23] (Fig. 2). The missense mutation can partially reveal the biochemical phenotype of AK deficiency and shows that *AK* gene inactivation causes a lack of AK activity. However, since this compound heterozygous mutation is connected to severe haemolytic anaemia, one could speculate that the resulting changes in red cell membrane structure may have some indirect effect on the function of the enzyme [9, 24]. In this study, we succeeded in diagnosing AK deficiency based on the decreased level of enzyme activity in red cells.

Recently, Dongerdiye et al. [13] reported a case of AK deficiency caused by two missense mutations in the *AK1* gene in a 6-year-old boy in India. These mutations included c.71A > G (p.Gln24Arg) in exon 4 and c.413G > A (p.Arg138His) in exon 6 (Table 1). In this case, the patient had clinical manifestations of severe haemolytic anaemia, without mental and psychomotor retardation. It is worth noting that one of the mutations in our present case is the same as in the Indian case: c.413G > A (p.Arg138His). Due to the rarity of this disease and two cases of the same mutation site, we speculate that this mutation site could be a high-frequency mutation type.

The C-WYCSI test suggested mild intellectual disability in the patient, who exhibited psychomotor impairment (Table 1). A few cases of intellectual disability in a patient with AK deficiency and severe haemolytic anaemia have been described [15]. The reduction in AK activity could be the reason for the neurologic impairment, although the mechanisms responsible are not well understood. Since the AK1 isoenzyme is expressed in red blood cells and the brain, the mutation may be accompanied by brain abnormalities [16, 17].

We conducted a literature review and made a speculation on the aspect of intellectual disability. AK1 expression is uniquely confined to neurons, including Purkinje cells in the cerebellum, and its transcript level is well correlated with enzymatic capacity [25]. Hu et al. [26] showed that AK1 is an autosomal recessive ID (ARID) in consanguineous families.

He also pointed out that missense mutations are a common type of mutation in inherited anaemic diseases caused by AK deficiency, including c.413G > A and c.286C > T, and may be related to other psychomotor development problems like autism spectrum disorder (ASD). Previous literature reports have shown that AK defects with an intermediate level of enzyme activity in the heterozygotes and severe deficiency in the homozygotes combined with other erythrocyte enzyme defects (G6PD) [27]. Boivin et al. [28] described intellectual disability in a patient with severe red cell AK deficiency and whose AK activity ranged between less than 1% and 13% of normal in 1971. However, this was attributed to the forceps delivery and resuscitation at birth. Patients with residual red cell AK activity that was only partially reduced (from 20 to 50% of normal) can only exhibit mild to moderate hemolytic anemia with normal mental development [17]. However, in the case from India with the same mutation site (p. Arg138His), AK enzyme activity was measured and found to be 63.0 IU/g Hb (297–360 IU/g Hb). This value was 21.2% of the normal reference range. In this case, after repeated transfusion therapy, AK activity detection was slightly below the normal range (92% of normal). Due to the early onset of the disease, severe heamolytic anaemia was present in the early stage of the disease. Thus, we could believe that the AK enzyme activity in our patient may be significantly lower than normal in the early stage. According to previous reports, we have reason to believe that intellectual disability may be positively correlated with AK enzyme activity.

Next, we mainly analyzed the frameshift mutation in this case. The frameshift mutation produced a random amino acid sequence downstream until a premature stop codon arises, which either leads to nonsense-mediated mRNA decay or truncation of the protein [29]. Genetic analysis showed the frameshift mutation site in this case, c.223dupA in exon 5, resulted in the emergence of a stop codon at amino acid site 93. This leads to the shortening of the peptide chain and the generation of nonfunctional peptide chain fragments. Such alterations ultimately lead to changes in pathogenicity. Some cases of blood system diseases have reported frameshift mutant could predict novel stop codons, that would lead to serious consequences [30, 31]. Hyejin et al. [32] explained a molecular mechanism by which AK1 may distort AMPK signalling by changing the nucleotide ratios under pathological conditions. Decreased AK1 protein enzyme activity causes AMPK dysregulation and thereby GSK3b activation, leading to neurodegeneration. Therefore, we speculated that a frameshift mutation was the main factor leading to the severe deficiency of AK enzyme activity in the early stage of this disease. The frameshift mutation (c.223dupA) may be the main reason for the significantly decreased AK1 activity. Therefore, this mutation may be related to the neuropathogenicity caused by AK1 deficiency. Long‐term observation of psychomotor impairment and further protein molecular signaling pathway detection are required to evaluate disease progression in future studies. This case also highlights the importance of next-generation sequencing as part of newborn to early childhood screening to diagnose rare and poorly diagnosed genetic diseases as early as possible.

## Supplementary Information

**Additional file 1**. **Supplementary Table S1:** Targeted NGS panel for the analysis for rare congenital anaemias.

## Data Availability

The datasets of the current study are not publicly available in order to protect participant confidentiality, but can be available from the corresponding author on reasonable request.

## Abbreviations

CNSHA: Chronic nonspherocytic haemolytic anaemia
ADP: Adenosine diphosphate
AMP: Adenosine monophosphate
ATP: Adenosine triphosphate
DNA: Deoxyribonucleic acid
AK: Adenylate kinase
G6PD: Glucose-6-phosphate dehydrogenase
PK: Pyruvate kinase
P5′N: Pyrimidine 5′-nucleotide
C-WYCSI: Chinese Wechsler Young Children scale of Intelligence
NGS: Next-generation sequencing
ARID: Autosomal recessive ID
ASD: Autism spectrum disorder
AMPK: Adenosine 5′-monophosphate-activated protein kinase
