# Supplementary figures and images for: Red cell adenylate kinase deficiency in China: molecular study of 2 new mutations (413G > A, 223dupA)
Source: BMC Med Genomics. 2022 May 4;15:102. doi: 10.1186/s12920-022-01248-2 (PMC9066714; doi:10.1186/s12920-022-01248-2)

## Slide 1
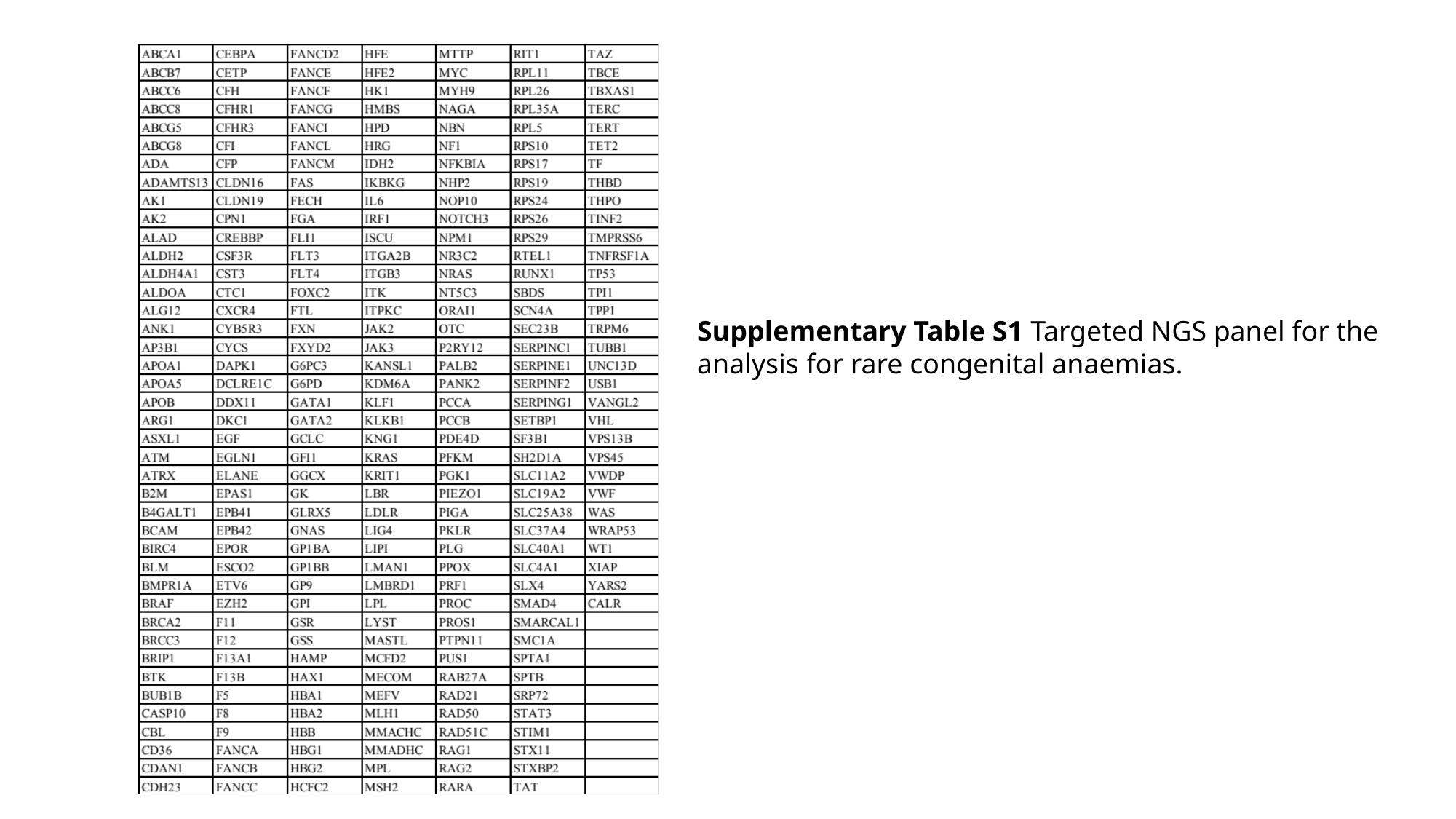

Supplementary Table S1 Targeted NGS panel for the analysis for rare congenital anaemias.

Supplement: Supplementary file 1 — Additional file 1. Supplementary Table S1: Targeted NGS panel for the analysis for rare congenital anaemias. [file 12920_2022_1248_MOESM1_ESM.pptx]
